# Complementary food hygiene practice and associated factors among mothers with children aged 6–23 months in Dessie Zuria, South Wollo Zone, Amhara, Ethiopia, 2023

**DOI:** 10.3389/fnut.2024.1465008

**Published:** 2024-11-01

**Authors:** Alemayehu Tesfaye Addis, Yeshimebet Ali Dawed, Geleta Mussa Yimer, Yonas Fissha Adem

**Affiliations:** ^1^Department of Public Health, Zemen Postgraduate College, Dessie, Ethiopia; ^2^Department of Public Health Nutrition, Wollo University, Dessie, Ethiopia; ^3^Department of Epidemiology and Biostatics, Wollo University, Dessie, Ethiopia; ^4^Department of Public Health, Dessie College of Health Sciences, Dessie, Ethiopia

**Keywords:** complementary feeding, hygiene practice, associated factors, children aged, 6–23 months

## Abstract

**Background:**

Implementing appropriate complementary food hygiene practices is essential to lower the incidence of food-borne disease and malnutrition in children. However, this aspect is often overlooked in resource-limited settings, and information regarding these practices is not fully available and is not assessed enough. Therefore, this study aimed to assess complementary food hygiene practices and their associated factors in Dessie Zuria, South Wollo Zone, Amhara, Ethiopia, in 2023.

**Methods:**

An institutional-based cross-sectional study design was conducted from 17 April to 18 May 2023, among 366 mothers with children aged 6–23 months. Dessie Zuria was purposively selected, and a systematic random sampling technique was used to recruit study participants. Data were collected using pretested and structured questionnaires. Finally, the data were entered using EPI-info and then exported to SPSS version 26. Bivariable and multivariable logistic regression analyses were used to identify factors associated with complementary feeding hygiene practices. Both crude odds ratios (COR) and adjusted odds ratios (AOR) with a 95% confidence level (CI) were computed, and a *p*-value of <0.05, in the final model, was considered statistically significant.

**Results:**

The proportion of households practicing complementary food hygiene was 22.22%. Factors associated with these practices included access to media such as television or radio (AOR = 10.51, 95% CI: 2.8, 39.28), starting complementary feeding before 6 months (AOR = 2.01, 95% CI: 1.05, 3.84), and the child’s age being 6 to 11 months (AOR = 0.25, 95% CI: 0.08, 0.7).

**Conclusion:**

The prevalence of complementary food hygiene practices was poor. Healthcare professionals should promote starting breastfeeding at the age of 6 months. In addition, media companies ought to make an effort to create a positive social and cultural environment that encourages complementary feeding practices for young children.

## Introduction

Complementary feeding begins when breast milk alone is no longer enough to meet a baby’s nutritional needs, and additional foods are required. In environments with limited resources, complementary foods can produce microbiologically hazardous diets that increase the risk of contracting food-borne infections (1). Food hygiene practices aim to prevent diseases associated with consuming contaminated food and water during the complementary feeding period (1).

Complementary feeding practices that are incorrect and unhygienic worldwide have been associated with malnutrition outcomes (wasting, stunting, and underweight) and under-five mortality. Previous studies have indicated that the timely introduction of complementary foods (CFs) to infants and young children (IYC) reduces the likelihood of malnutrition, infectious diseases, and mortality (2). According to the WHO and UNICEF, breastfeeding should continue until infants are at least 2 years old, after which they should begin eating a healthy diet (3). In addition, the WHO warns against starting CFs too soon or too late. IYC are more prone to diarrheal illnesses and subsequent malnourishment due to their early exposure to microbiological pathogens and the consumption of potentially hazardous or contaminated complementary foods and drinks (4, 5).

Contamination of food during the complementary phase is a major cause of diarrheal diseases in both the developed and developing world (6). If the hygienic conditions of complementary feeding are not correctly controlled, it can result in diarrhea and months of developmental retardation, which can lead to kwashiorkor, marasmus, and immunodeficiency characterized by recurring and chronic illnesses that can be fatal (7).

An average of 65% of deaths caused by diarrheal diseases could be reduced if good hygiene practices were accompanied by the provision of clean water and proper sanitation (8). Children’s growth and development greatly depend on eating a healthy diet, especially during their first 2 years of life. Every year, approximately 10 million children die, with 41% of these deaths occurring in sub-Saharan Africa and 34% in South Asia (9). The practice of complementary feeding (CF) is linked to detrimental, multifaceted effects on one’s health and development. It is the primary cause of over two-thirds of child mortality under five in Sub-Saharan Africa (10). Furthermore, poor food hygiene practices are linked to child undernutrition, and any harm resulting from nutritional deficiencies in early childhood is linked to impaired cognitive development, low educational attainment, and low economic output (11).

Since the majority of African nations have many socioeconomic and cultural characteristics in common, it is critical to pinpoint the causes of poor complementary feeding practices that are prevalent in these nations. Targeting individuals, families, and communities susceptible to inadequate hygiene practices related to complementary feeding (CF) is crucial for designing successful nutritional interventions. This approach focuses on identifying modifiable characteristics that are consistent across many developing nations (12).

According to population-based studies in West Africa, due to inadequate breastfeeding and other feeding hygiene practices, children between the ages of 3 and 15 months are most at risk for nutritional deficiencies and developmental retardation (13).

Poor feeding habits were caused by a lack of information about proper food handling, poor sanitation, and inadequate social amenities such as kitchens, sewage systems, and toilets (8). The causes of diarrheal illnesses extend beyond medical factors; social, economic, environmental, and behavioral facets of the family unit play a significant role. Studies conducted in Ethiopia, including those in Amhara (1), Harare (9), and Jigjiga, highlight these facets (7).

Significant effort has been dedicated to emphasizing the importance of maintaining proper hygiene when preparing complementary foods, ensuring enough food for the entire household, and having an appropriate understanding of nutrition (14). However, the application of these requirements during complementary feeding has not been adequately addressed. In addition, there is a problem with real hygiene practices of complementary feeding, resulting in serious consequences of poor child health outcomes. Therefore, this study aimed to explore complementary food hygiene practices and their associated factors among mothers with children aged 6–23 months in Dessie Zuria Woreda, South Wollo Zone, Amhara region, Ethiopia, 2023.

## Materials and methods

### Study design, area, and period

An institutional-based cross-sectional study design was conducted from 28 April 28 to 7 June 2023, in the Dessie Zuria district, which is located in the South Wollo Zone of Amhara Regional State. It is bordered by Albuko and Wereilu to the south; Tenta to the northwest; Kutaber to the north; Tehuledere to the northeast; and Kalu to the east. There are 32 kebele in the area, housing a total of 201,000 people. There are 8 governmental health centers, and 32 health posts exist in the study area. Health centers were provided Stabilization Centers (SC), and health posts provided Outpatient Therapeutic Programme (OTP) complementary feeding.

#### Source population

All lactating mothers with children aged 6–23 months who attended the health institutions of the Dessie Zuria district were the source population.

#### Study population

The study population included all lactating mothers who visited the selected health institutions and who had children aged 6–23 months during the study period.

#### Eligibility criteria

##### Inclusion criteria

- Mothers whose children were between the ages of 6 and 23 months were included.

##### Exclusion criteria

- Mothers who were seriously ill and unable to hear were not included in the study.

### Sample size determination

The sample size was calculated using the single population proportion formula, based on the following assumptions: a 95% confidence level (CI), a margin of error (D = 0.05), and a percentage of hygienic practices among mothers of infants aged 6 to 23 months during complementary feeding, which was found to be 38.9% in a previous study conducted in Bahir Dar, Amhara (1).


$$n=\frac{Z^{2}a/2\times P\left(1-P\right)}{d^{2}}$$

where *n* = the required sample size,

*Z* = a standard score corresponding to a 95% confidence level;

*P* = proportion of hygienic practice among mothers with children aged 6–23 months during complementary feeding found = 38.9%.

*d* = margin of error = 0.05.


$$n=\frac{{\left(1.96\right)}^{2}\times 0.389\left(0.611\right)}{{\left(0.05\right)}^{2}}=366$$

then, the total required sample size was 366.

### Sampling procedure

From a total of 40 (32 health posts and 8 health centers) facilities providing health services in the Dessie Zuria district, 15 (11 health posts and 4 health centers) facilities were selected using simple random sampling. Sample sizes were proportionally allocated to each health facility, including health centers and health posts, and a systematic random sampling technique was used to recruit the study participants. Considering N (the total number of women who came for their complementary feeding in the previous 2 months at the health facility = 472), n (the calculated sample size = 366), and the k-interval (where *K* = *N*/*n* = 472/366 = 2), the first participant was selected using a lottery method among the first two complementary feeding users at each health center and health post.

### Variables

#### Dependent variable

Complementary food hygiene practices (categorized as poor or good).

#### Independent variables

- Socio-economic and demographic characteristics: Age, marital status, family size, educational status, husband’s educational status, occupational status, and family wealth.
- Maternal health service and related characteristics: Parity, ANC visit, place of delivery, PNC visit, knowledge, and access to mass media.
- Components of hygienic practice: a latrine, mother hand washing with soap after using the toilet, hand washing with soap before feeding and food preparation, access to a private latrine, feeding of leftovers, washing of utensils, serving cooked food immediately for children, food cooking type, place of food preparation and its modernity, water supply, and source/access to information media such as television.
- Child-related characteristics: child age, sex, birth order, and breastfeeding.

### Operational definitions

- Food hygienic practice is defined as the feeding practice(s) of mothers when feeding their children based on the latest WHO recommendations among the WHO feeding practice indicators (15).
- Complementary feeding is the period (between 6 months and 2 years) during which foods or liquids are provided along with continued breastfeeding (16).
- Complementary food hygiene practices are a collection of fundamental guidelines used to systematically regulate the environmental conditions during manufacture, storage, and use when providing children between the ages of 6 and 23 months with complementary feeding. Six questions regarding hand washing with soap and water and three scales were used to quantify it: 1—always, 2—sometimes, and 3—wash only with water; 10 questions with a “yes” or “no” response on safety precautions to take when preparing food. The responses forwarded by the study participants to the 6 questions related to hand washing with water and soap were dichotomized as 1 “for always” and 0 “for sometimes and washing only with water.” The responses forwarded by the study participants to 10 questions related to safety measures during food preparation were dichotomized as 1 “for yes” and 0 “for no responses.” Study participants were classified as having good hygiene practices during complementary feeding if they correctly answered 75% of the questions; if not, they were classified as having poor hygiene practices during complementary food feeding (14).

### Data collection procedure

A structured questionnaire that included questions that assessed the study variables was prepared and adapted from different literature. The questionnaire consisted of demographic data and complementary feeding practice tools. The data were collected by four healthcare workers and were supervised by the principal investigator.

### Data quality control measure

The relevant literature was reviewed, and a structured validated questionnaire was created in English, translated into Amharic, the local language, and then back into English to guarantee consistency. A pretest was conducted for 5% of the sample size both in health centers and health posts where actual data were not collected. After that, appropriate modifications were made before the actual data collection. Training data collectors, tight monitoring, timely feedback, and daily assessment of all completed questionnaires were used to ensure the quality of the data. Before beginning the data collection, data collectors received a 1-day training covering the administration of each question and ethical guidelines. The supervisor monitored the data every day to ensure its accuracy and completeness.

### Data analysis and management

The data were coded and entered using Epi-Data version 4.6 software. The data were exported to SPSS version 26 for further statistical analyses. Descriptive and analytical analyses were also performed. For continuous data, descriptive features were expressed as mean (standard deviation), median (interquartile range), and frequency distribution for categorical data. Frequency tables, graphs, and cross-tabulations were used to present the findings of the study. Both bivariable and multivariable logistic regression models were used to identify factors associated with complementary feeding hygiene practices, and those variables with a *p*-value of ≤0.25 during bivariable analysis were entered into the multivariable analysis. Adjusted odds ratios with 95% confidence level were computed to assess the association between independent predictors and outcome variables. Then, the significance association was declared at a *p*-value of <0.05.

## Results

### Socio-demographic characteristics

A total of 351 mothers with children aged 6–23 months participated in this study, with a response rate of 96%. The mean age of the mothers was 28.54, with a standard deviation of ±0.25 years and (95% CI of 28.04, 29.03). A total of 80 (22.79%) and 92 (26.21%) of mothers were government employees and merchants, respectively. For fathers, 142 (22.79%) and 84 (26.21%) were merchants and daily laborers, respectively (Table 1).

**Table 1:** Socio-demographic characteristics of the CFHP participants in the Dessie Zuria health institutions, Amhara, Ethiopia, 2023 (*n* = 366).

Variables	Groups	Frequency	Percent
Resident	Urban	41	11.7
	Rural	310	88.3
Educational status	Unable to read and write	21	5.98
	Able to read and write	198	56.4
	Grades 9–12 complete	31	8.83
	Diploma	18	5.13
	First-degree and above	83	23.7
Occupational status of mothers	Government employee	80	22.8
	Merchant	92	26.2
	Daily labor	68	19.4
	Urban agriculture	14	3.99
	Housewife	59	16.8
	Other	38	10.8
Occupational status of fathers	Government employee	51	14.5
	Merchant	142	40.5
	Daily labor	84	23.9
	Urban agriculture	46	13.1
	Other	28	7.98
Access to media	Yes	102	29.1
	No	249	70.9
Child age	6–11	228	65
	12–23	123	35
Child sex	M	180	51.3
	F	171	48.7
Household private latrine	Yes	73	20.8
	No	278	79.2

### Maternal, child, and service-related characteristics of respondents

Among the participating children, 236 (67.24%) started their complementary feeding at the age of 6 months; during this data collection, 244 (69.52%) of children were not currently breastfeeding. Among the total respondents, only 69 individuals (19.66) were cleaning food utensils with hot water. Similarly, 82 (23.36%) participants were using soap or ash for washing food utensils. Of the total study participants, 209 (59.54%) reported that their household’s source of drinking water was piped water (Table 2).

**Table 2:** Services for complementary food hygienic practices demonstrated among children aged 6–23 months in the Dessie Zuria health institutions, Amhara, Ethiopia.

Variables	Groups	Frequency	Percent
Age of starting complementary feeding	Before 6 months	61	17.4
	At 6 months	236	67.2
	Later after 6 months	54	15.4
Currently breastfeeding	Yes	107	30.5
	No	244	69.5
Use of hot water for cleaning food utensils	Yes	69	19.7
	No	282	80.3
Use soap or ash for washing food utensils	Yes	82	23.4
	No	269	76.6
Source of drinking water	Piped water	209	59.5
	Well water	54	15.4
	River water	68	19.4
	Spring water	20	5.7
Water treatment options	Chlorine	148	42.2
	Water treatment solution	96	27.4
	Boiling	69	19.7
	No usage of water treatment	38	1.83
Food groups in the last 24 h	Dairy products	152	43.3
	Eggs	43	12.2
	Grains, roots, and tubers	27	7.69
	A-rich fruits and vegetables	24	6.84
	Other fruits and vegetables	47	13.4
	Meat, poultry, fish, and shellfish	18	5.13
	Legumes and nuts	24	6.84
	Foods cooked with fats	16	4.56
Consumed food groups	Consumed 0–2 food groups	213	60.7
	Consumed 3–4 food groups	138	39.3

### Food hygienic practice

**Figure 1:**
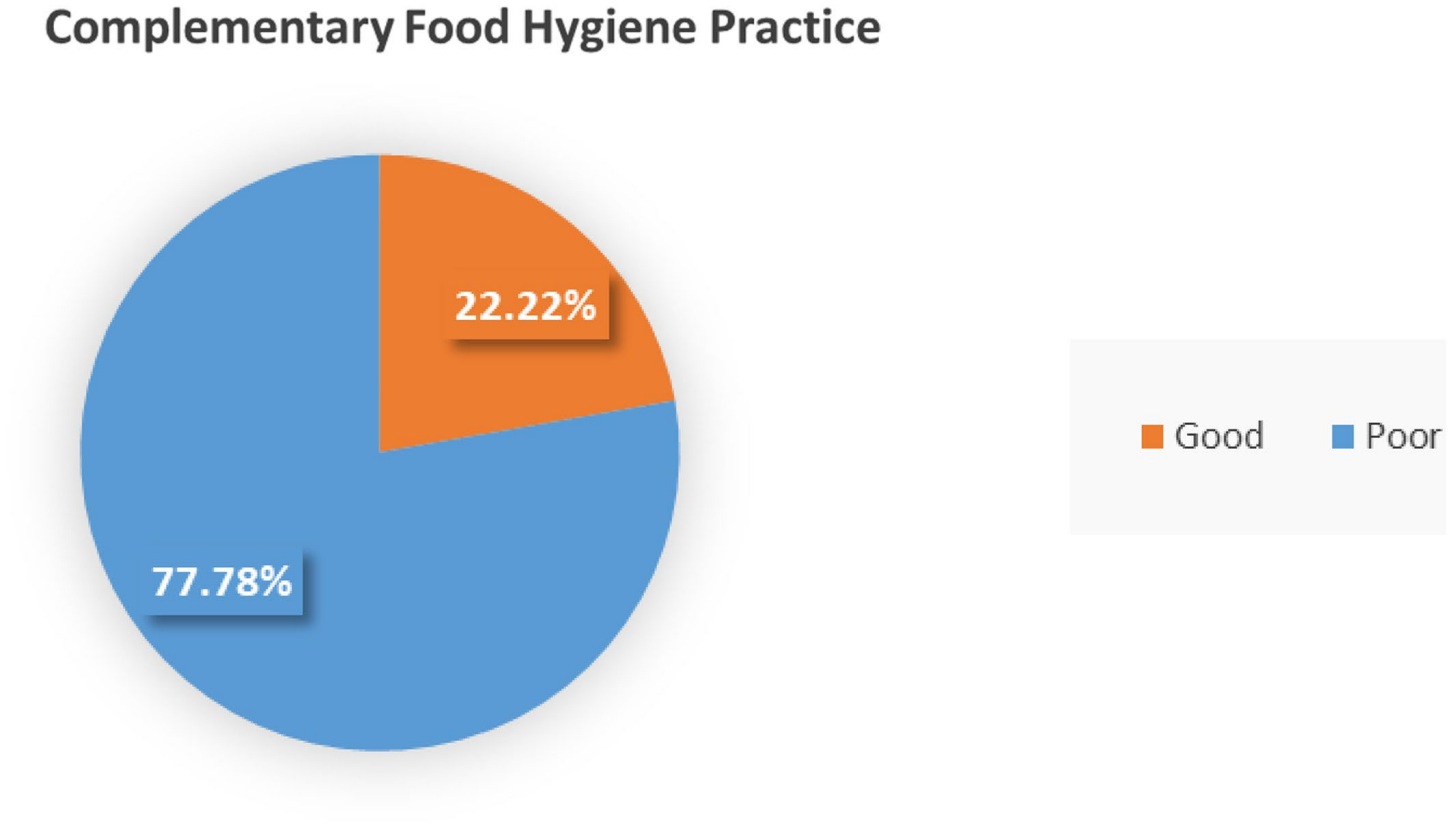
Complementary food hygienic practice among mothers who had children aged 6—23 months in the Dessie Zuria health institutions, Amhara, Ethiopia.

The current complementary food hygienic practice was 78 (22.22%) among mothers with children aged 6–23 months according to this research finding (Figure 1).

Complementary food hygienic practices among mothers with children aged 6–23 months were evaluated based on 6 handwashing indicators and 10 questions related to safety measures during food preparation, according to the following responses (Table 3).

**Table 3:** Complementary food hygienic practice questionnaire among mothers with children aged 6–23 months in the Dessie Zuria health institutions, Amhara, Ethiopia, 2023.

Variables	Groups	Frequency	Percent
Mothers hand washing with soap after using the toilet	Always water with soap	213	60.7
	Not always water with soap	80	22.8
	Wash only with water	36	10.3
	Do not wash	22	6.27
Mothers hand washing with soap before feeding the child	Always water with soap	236	67.2
	Not always water with soap	81	23.1
	Wash only with water	34	9.69
Hand washing with soap before food preparation	Always water with soap	230	65.5
	Not always water with soap	71	20.2
	Wash only with water	50	14.3
Washing of utensils	Always water with soap	219	62.4
	Not always water with soap	82	26.2
	Wash with ash	40	11.4
Mother’s fingernails cut short	Yes	76	21.7
	No	275	78.4
Children hand washing after using the toilet	Always water with soap	213	60.7
	Not always water with soap	94	26.8
	Do not wash	44	12.5
Children hand washing before eating	Always water with soap	227	64.7
	Not always water with soap	57	16.2
	Did not wash	67	19.1
Serving cooked food immediately	Yes (if served within two 2 h)	226	64.4
	No (if not served within two 2 h)	126	35.6
Serving leftover food for children.	Always serving leftover food	147	41.9
	Sometimes serving leftover food	104	29.6
	Never serving leftover food	100	28.5
Presence of children’s private feeding utensils	Yes	67	19.1
	No	284	80.9
Children’s clean feeding utensils	Yes	269	76.6
	No	823	23.4
Separating utensils for raw and cooked food	Yes	100	28.5
	No	251	71.5
Place of food cooking preparation	Cultural stove	225	64.1
	Modern stove	126	35.9
Keeping ready-to-eat food	Always	235	67
	Not always	116	33.1
HH has a hand washing facility after a toilet visit	Yes	80	22.8
	No	271	77.2
Hand washing facility has soap or ash with water	Yes	67	19.7
	No	206	80.3

### Factors associated with complementary food hygienic practices

The bivariable analysis showed that resident, occupational status of mothers, occupational status of fathers, access to media, child age, household private latrine, currently breastfeeding, age of starting complementary feeding, use of hot water for cleaning food utensils, source of drinking water, and water treatment options were selected candidate variables for the multivariable analysis.

In the final multivariable logistic regression analysis, this study found that the presence of a private latrine (AOR = 0.13, 95% CI: 0.021, 0.87), starting complementary feeding before 6 months (AOR = 8.30, 95% CI: 1.15, 59.81), and starting complementary feeding at 6 months (AOR = 9.10, 95% CI: 1.16, 71.21) were found to be associated factors of complementary food hygienic practices at the p-value of less than 0.05 (Table 4).

**Table 4:** Factors associated with complementary food hygienic practices among women with children aged 6–23 months in the Dessie Zuria health institutions, Amhara, Ethiopia, 2023.

Variables/categories	Complementary food hygienic practice		COR (95% CI)	AOR (95% CI)
	Good	Poor		
Resident				
Urban	14	27	3.11(1.68–5.77)	0.61(0.25–1.45)
Rural	64	246	1	1
Occupational status of mothers				
Government employee	9	72	3.13(1.37–7.13)	2.48(0.68–8.94)
Merchant	12	69	5.52(2.38–12.78)	1.72(0.45–6.56)
Daily labor	15	42	0.88(0.10–7.85)	2.25(0.68–7.39)
Urban agriculture	11	9	1.83(0.70–4.75)	0.98(0.28–3.50)
House wife	13	48	0.24(0.02–1.99)	1.13(0.19–6.61)
Other	18	33	1	1
Occupational status of fathers				
Government employee	17	57	1.97(0.76–5.01)	0.37(0.07–1.81)
Merchant	27	51	4.32(1.64–11.37)	0.69(0.15–3.17)
Daily labor	28	116	2.43(0.89–6.66)	0.99(0.22–4.51)
Urban agriculture	6	49	1	1
Access to media				
Yes	6	96	6.51(2.73–15.52)	10.51(2.8–39.28)*
No	72	177	1	1
Child age				
6–11	63	165	0.36(0.19–0.67)	0.25(0.08–0.7) *
12–23	15	108	1	1
Household private latrine				
Yes	12	61	1.58(0.80–3.11)	1.62(0 0.69–3.81)
No	66	212	1	1
Currently breastfeeding				
Yes	88	19	1.47(0.83–2.62)	0.61(0.25–1.47)
No	185	59	1	1
Age of starting complementary feeding				
less than 6 months	35	88	1.71(1.02-2.85)	2.01(1.05-3.84) *
6 months and above	43	185	1	1
Use of hot water for cleaning food utensils				
Yes	10	59	1.87(0.90–3.86)	0.25(0.04–1.45)
No	68	214	1	1
Source of drinking water				
Piped water	56	152	0.219(0.07–0.62)	0.44(0.08–2.44)
Well water	5	50	0.52(0.25–1.07)	3.35(0.78–14.38)
River water	10	57	1.359(0.52–3.53)	2.93(0.86–9.87)
Spring water	7	14	1	1
Water treatment options				
Chlorine	43	108	0.46(0.24–0.88)	0.62(0.25–1.51)
Water treatment solution	16	80	0.54(0.27–1.09)	0.51(0.14–1.87)
Boiling	13	56	0.24(0.07–0.03)	0.52(0.13–2.02)
No usage of treatment	6	29	1	1

COR, crude odds ratio; AOR, adjusted odds ratio; CI, confidence interval. *p-value *< 0.05*.

## Discussion

In this study, the proportion of hygienic practice of complementary food among mothers with children aged 6–23 months who lived in Dessie Zuria was 22.22, 95% CI: (17.85–26.59). The prevalence of hygienic complementary food feeding practices in this study was lower than that reported in previous studies conducted in Bahir Dar (38.9%) (1), Harare (39.6%) (9), Jigjiga (7), Debark (14), and Bangladesh (15). This difference could be due to the study setting; previous studies were conducted in both rural and urban communities, while this study was conducted solely in a rural area. Consequently, women in this rural setting may have less access to information about hygienic practices, which could be the reason for a lower prevalence of hygienic complementary food practices than the previous findings. In addition, complementary food hygienic practices are affected by a range of climatic conditions and socio-political statuses.

Concerning the factors, household access to media, such as TV or radio, was a significant predictor of hygienic complementary food feeding practices among mothers with children aged 6–23 months. The odds of hygienic complementary food feeding practices were higher in mothers who had access to media, such as TV or radio, than in mothers who did not have access to media (AOR = 10.51, 95% CI: 2.8, 39.28). A similar finding was reported in a study conducted in Antsokia Gemza district, Wolaita Sodo town, and Bangladesh (17–19). This may be due to the fact that the media is vital in providing knowledge regarding complementary feeding techniques, which is necessary for their adoption. In addition, mothers and other caregivers who have access to the media are probably more knowledgeable and adhere to proper hygienic practices when it comes to complementary feeding. Thus, they adhere to specific habits including maintaining their diet, cutting their fingernails, and washing their hands before cooking complementary foods.

Starting complementary feeding before 6 months was significantly associated with complementary food hygiene practices. The odds of complementary food hygiene practices were 2 times more than starting complementary feeding after 6 months (AOR = 2.01, 95% CI: 1.05, 3.84). This finding is contrary to studies using secondary data analysis from the Ethiopian Mini-Demographic and Health Survey 2019, in which the age of starting complementary feeding was not significantly associated with complementary food hygienic practices (20). The possible explanation for this difference is that mothers may pay more attention to hygiene when starting complementary feeding before 6 months, and later on, they may reduce these hygiene precautions.

In the present study, children aged 6 to 11 months were less likely to be practicing complementary food hygiene as compared to a child aged 12–23 months (AOR = 0.25, 95% CI: 0.08, 0.7). This result is supported by studies conducted in Tigray, Western Ethiopia, and Nigeria (21–23). The reason may be that younger children receive suboptimal complementary feeding since family foods are introduced to them later than they are to older children, who then incorporate them into their meals. In other words, older children’s food consumption more readily reflects the nutritional diversity of the home than does the dietary intake of younger children. In addition, younger children are more likely to have fewer eating occasions, which could make it more difficult to achieve the requirements for complementary feeding. Mothers might also believe that their younger children’s digestive systems are still developing, leading them to avoid or omit specific foods from their meals, such as fats or meat products.

## Strengths and limitations of the study

A major strength of the present study is the use of the latest WHO recommendations on complementary feeding practices. In addition, the data were collected only from respondents who were on complementary feeding practices. However, the findings of this study were not triangulated with qualitative findings; in addition, the study presented self-reported data, which could be prone to social desirability and recall bias.

## Conclusion

The prevalence of good hygienic practices during complementary food feeding among mothers with children aged 6–23 months was low in this study area. This study also revealed that household access to media, such as television or radio, starting complementary feeding before 6 months, and children aged 6 to 11 months had a statistically significant association with complementary food hygienic practices. It is recommended that the Dessie Zuria health office and institutions work on enhancing health posts and training health extension workers to increase the prevalence of complementary food hygienic practices. Additionally, efforts should be made to promote the start of breastfeeding at the age of 6 months. In addition, governments, health professionals, and media outlets should collaborate to create and disseminate evidence-based messages to successfully reach the target population. Healthcare workers need to strengthen counseling mothers during growth monitoring sessions on complementary food handling.

## Data Availability

The raw data supporting the conclusions of this article will be made available by the authors, without undue reservation.
